# Inhibition of Chemokine-Glycosaminoglycan Interactions in Donor Tissue Reduces Mouse Allograft Vasculopathy and Transplant Rejection

**DOI:** 10.1371/journal.pone.0010510

**Published:** 2010-05-06

**Authors:** Erbin Dai, Li-Ying Liu, Hao Wang, Dana McIvor, Yun ming Sun, Colin Macaulay, Elaine King, Ganesh Munuswamy-Ramanujam, Mee Yong Bartee, Jennifer Williams, Jennifer Davids, Israel Charo, Grant McFadden, Jeffrey D. Esko, Alexandra R. Lucas

**Affiliations:** 1 Vascular Biology Research Group, Robarts Research Institute, The University of Western Ontario, London, Ontario, Canada; 2 Departments of Medicine and Surgery, and Microbiology and Immunology, The University of Western Ontario, London, Ontario, Canada; 3 Division of Cardiovascular Medicine, University of Florida, Gainesville, Florida, United States of America; 4 Department of Molecular Genetics and Microbiology, University of Florida, Gainesville, Florida, United States of America; 5 Viron Therapeutics, Inc., London, Ontario, Canada; 6 Gladstone Institute, San Francisco, California, United States of America; 7 Department of Cellular and Molecular Medicine, University of California San Diego, San Diego, California, United States of America; Charité-Universitätsmedizin Berlin, Germany

## Abstract

**Background:**

Binding of chemokines to glycosaminoglycans (GAGs) is classically described as initiating inflammatory cell migration and creating tissue chemokine gradients that direct local leukocyte chemotaxis into damaged or transplanted tissues. While chemokine-receptor binding has been extensively studied during allograft transplantation, effects of glycosaminoglycan (GAG) interactions with chemokines on transplant longevity are less well known. Here we examine the impact of interrupting chemokine-GAG interactions and chemokine-receptor interactions, both locally and systemically, on vascular disease in allografts.

**Methodology/Principal Findings:**

Analysis of GAG or CC chemokine receptor 2 (CCR2) deficiency were coupled with the infusion of viral chemokine modulating proteins (CMPs) in mouse aortic allograft transplants (n = 239 mice). Inflammatory cell invasion and neointimal hyperplasia were significantly reduced in N-deacetylase-N-sulfotransferase-1 (*Ndst1* ^f/f^ *TekCre* ^+^) heparan sulfate (GAG)-deficient (*Ndst1^−/−^*, p<0.044) and CCR2-deficient (*Ccr2^−/−^*, p<0.04) donor transplants. Donor tissue GAG or CCR2 deficiency markedly reduced inflammation and vasculopathy, whereas recipient deficiencies did not. Treatment with three CMPs was also investigated; Poxviral M-T1 blocks CC chemokine receptor binding, M-T7 blocks C, CC, and CXC GAG binding, and herpesviral M3 binds receptor and GAG binding for all classes. M-T7 reduced intimal hyperplasia in wild type (WT) (*Ccr2^+/+^*, p≤0.003 and *Ccr2^−/−^*, p≤0.027) aortic allografts, but not in *Ndst1^−/−^* aortic allografts (p = 0.933). M-T1 and M3 inhibited WT (*Ccr2^+/+^* and *Ndst1* ^+/+^, p≤0.006) allograft vasculopathy, but did not block vasculopathy in *Ccr2^−/−^* (p = 0.61). M-T7 treatment alone, even without immunosuppressive drugs, also significantly prolonged survival of renal allograft transplants (p≤0.001).

**Conclusions/Significance:**

Interruption of chemokine-GAG interactions, even in the absence of chemokine-receptor blockade, is a highly effective approach to reduction of allograft rejection, reducing vascular inflammation and prolonging allograft survival. Although chemokines direct both local and systemic cell migration, interruption of inherent chemokine responses in the donor tissue unexpectedly had a greater therapeutic impact on allograft vasculopathy.

## Introduction

Chemokines interact with both glycosaminoglycans (GAG) and cell surface receptors. Both interactions accelerate and localize inflammatory cell responses in damaged or transplanted tissues, but how the interaction of chemokines with tissue polysaccharides, particularly GAGs, regulate pathologic inflammatory responses is only partially understood [1]–[4]. The relative impact of GAG versus receptor interactions with chemokines on the progression of transplant rejection is not known. Chemokines are small 8–12 kDa proteins, organized into C, CC, CXC, and CX3C subclasses; the CC class is traditionally defined as directing monocyte and lymphocyte activation and the CXC class as directing neutrophil activation [1]–[3]. There is, however, extensive crossover of receptor and cellular targets. Chemokines oligomerize on GAGs to form three-dimensional concentration gradients that attract cells to sites of tissue damage, such as in a fresh organ transplant. These GAG-chemokine interactions are postulated to increase the specificity of chemokine-directed chemotaxis of innate immune cells when combined with receptor recognition [1]–[3]. Binding to tissue and cell surface GAGs is thought to present the chemokine N-terminus to inflammatory cell surfaces where the cognate seven-transmembrane G-protein coupled chemokine receptors initiate the signaling responses that drive leukocyte taxis. These interactions directionally guide cells toward increasing concentrations of chemokines and act as a primary defense to remove pathogens or to begin repair responses after trauma, transplant, or injury [1]–[5]. Chemokines are also reported to direct trafficking of dendritic cells [6] and lymphocytes [6]–[9] in bone marrow and secondary lymphoid organs [lymph nodes, tonsil], as well as at local tissue sites.

Transplant vasculopathy is a form of highly inflammatory chronic transplant rejection that is one of the leading causes of organ loss after the first year post transplant [10]–[12]. Up to 50% of late transplant loss has been attributed to early damage and innate immune or inflammatory, reactions (non acquired immune) that are up-regulated in response to transplanted organ ischemia, increased inflammatory cytokine expression in the donor and surgical trauma.

Both acute and chronic rejection, as well as accelerated transplant vasculopathy, are associated with increased chemokine and chemokine receptor expression, including increased macrophage chemoattractant protein-1 (MCP-1, CCL2), macrophage inflammatory protein-1α (MIP1α, CCL3), regulated on activation T cell expressed and secreted (RANTES, CCL5), CCR2, and CXCR3 among others [13]–[18]. Inflammatory cell recruitment, vascular diseases, and rejection are reduced in mice with selected deficiencies for certain chemokines or their receptors or after treatment with inhibitory reagents that target chemokines such as MCP-1 (CCL2)[19], CCR1 [20], CCR2 [19], CCR5 [21], CX3CR1 [22], and CXCR3 [22]. The effects of inhibition or down-regulation of individual chemokines in transplant rejection can vary. For example, while atherosclerosis and rejection of pancreatic islet cell transplants are consistently reduced in CCR2 deficient mice, transplant rejection was only minimally altered after heart transplant into *Ccr2* ^−/−^ recipients [19]–[23].

The distribution of GAGs in the arterial wall also correlates with vascular disease [3]. Heparan sulfate (HS), chondroitin sulfate, dermatan sulfate, keratan sulfate, and hyaluronic acid are present in the arterial wall on cells and interstitial connective tissue areas, typically as proteoglycans [3], [24]. Increased circulating GAGs are detected in patients with acute renal transplant rejection, which may reflect local degradation of proteoglycans [25]. The importance of chemokine-Heparan sulfate interactions was recently demonstrated in mice bearing a tissue-specific mutation in the biosynthetic enzyme N-deacetylase/N-sulfotransferase-1 (*Ndst1*), which reduces the overall sulfation of HS chains [4]. Selectively inactivating the gene in endothelial cells and leukocytes reduced inflammatory neutrophil cell invasion with deficiency for chemokine-mediated transcytosis across cell layers. Based on bone marrow transplantation, the phenotype was associated with *Ndst1*-deficiency (*Ndst1^−/−^*) in endothelial cells, whereas deficiency in leukocytes had minimal effect [4]. Selectively inactivating the gene in endothelial cells and leukocytes reduced inflammatory neutrophil cell invasion. Based on bone marrow transplantation experiments, the phenotype was associated with *Ndst1*-deficiency in endothelial cells, whereas deficiency in leukocytes had minimal effect [4]. Chemokine binding to HS was also significantly reduced in *Ndst1* ^−/−^ endothelial cells. Mutating the GAG binding epitopes of chemokines also blocks cell migration *in vivo* in a mouse peritoneal cell migration assay [26]. Conversely, HS has been reported to prolong transplant function, reduce rejection [27] and modify xenograft vasculopathy, suggesting an alternate anti-inflammatory and protective role [28]. The role of GAGs and specifically GAG-chemokine interactions in transplant vasculopathy and rejection is not well understood.

Viral chemokine modulating proteins (CMPs) have highly active anti-inflammatory functions that have evolved in large DNA viruses over many millions of years. These CMPs can selectively target both chemokine-GAG and chemokine-receptor interactions [29]–[31]. M-T1 and M-T7 are secreted myxoma viral (rabbit) CMPs; M-T1 interferes with receptor binding of CC chemokines, and M-T7 interferes with GAG binding for C, CC, and CXC chemokines [31]–[34]. M-T7 also binds interferon gamma (IFNγ), but only inhibits the rabbit ligand in a species-specific fashion [35]–[37]. M3 is a secreted γ68 herpesvirus protein that blocks both receptor and GAG binding of C, CC, CXC, and CX3C chemokines and reduces inflammatory cell invasion in mouse herpes meningitis [38]. When infused as purified protein at the time of aortic transplantation, M-T1, M-T7, and M3 significantly reduced plaque growth in rat ACI to Lewis aortic allografts at 4 weeks [39]. Similarly, reduced vasculopathy and scarring were observed in renal transplants at 5 months after 10 daily injections of M-T7 together with cyclosporine, but without effects on overall survival [40]. Reductions in inflammatory cell invasion and plaque growth followed M-T7 treatment in both rat and rabbit iliofemoral angioplasty balloon injury models, indicating that M-T7 anti-inflammatory activity is not IFNγ dependent but instead is postulated to function through chemokine modulation, in a species independent fashion [41].

In this study, we examine the effects of chemokine-GAG interactions on inflammatory cell responses, vasculopathy development, and graft survival in mouse aortic and renal transplant models, with comparison to chemokine-receptor interactions. We have detected significant local reductions in inflammatory cell responses and vasculopathy development with either GAG or CCR2 deficiency or after treatment with viral chemokine modulating proteins (CMPs) targeting GAG or receptor binding. Abdominal aortic transplants from donor mice deficient in HS-GAG (*Ndst1^−/−^*) or the chemokine CCR2 receptor (*Ccr2^−/−^*) displayed marked reductions in plaque growth while HS GAG or CCR2 deficiency in the recipient mouse had minimal effects. Similarly, mice treated with CMPs also had markedly reduced inflammation and vasculopathy after aortic transplant. M-T7 treatment alone further reduced inflammatory cell invasion and prolonged survival in mouse renal allograft transplants, even in the absence of cyclosporine treatment.

## Results

### Deficiency of either Ndst1 or Ccr2 in aortic donor segments reduces inflammation and vasculopathy after aortic transplant in mouse models

**Figure 1:**
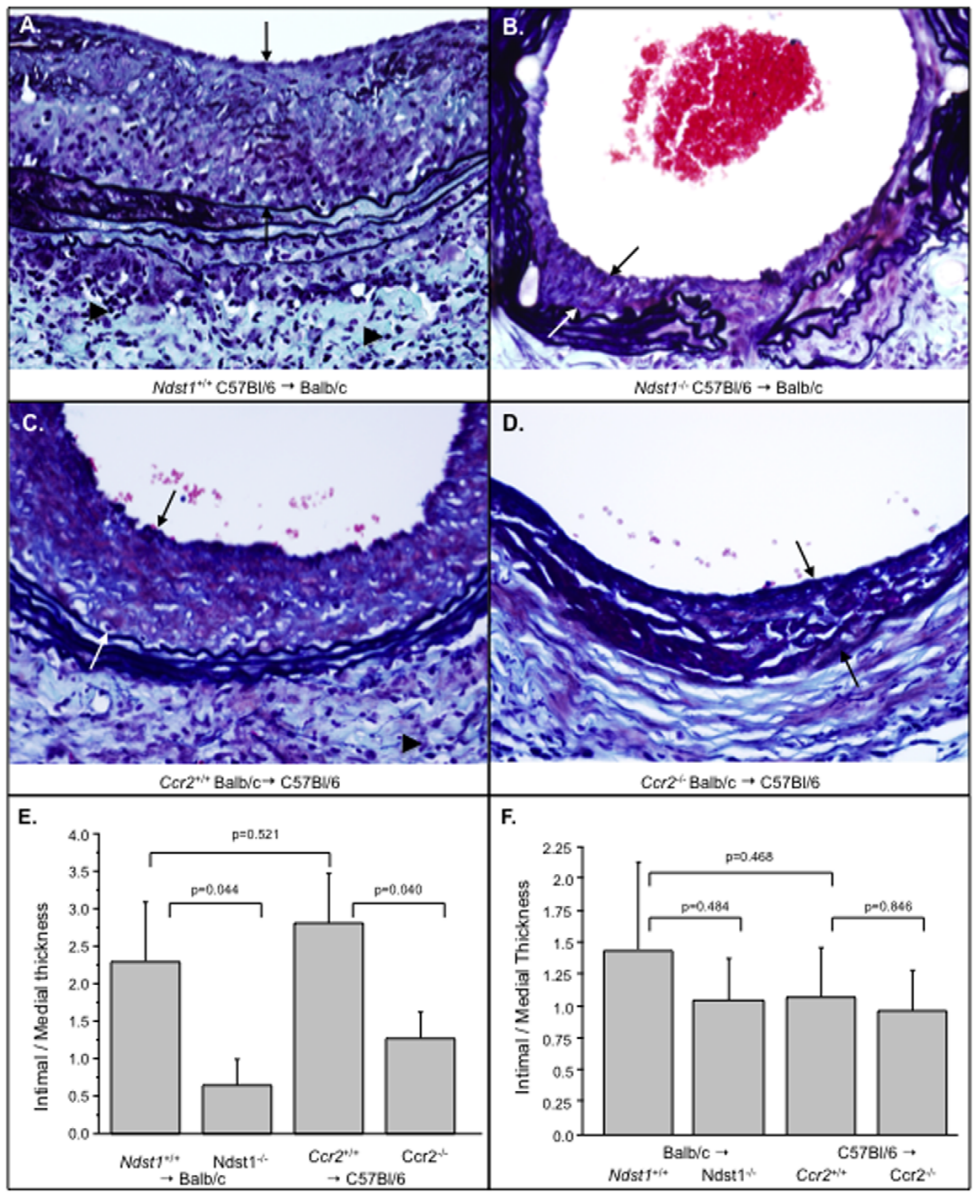
Local, but not systemic, GAG or receptor deficiency reduces neointimal hyperplasia in mouse aortic allograft transplants. Histology cross sections of aortic allograft transplants stained with Masson's trichrome demonstrating reduced inflammatory cell invasion with *Ndst1*-deficient (*Ndst1* ^−/−^) (**B**) and *Ccr2*-deficient (*Ccr2* ^−/−^) (**D**) donor aortic allografts when compared to wild type (WT)(**A**, **B**). Bar graphs demonstrate similar, significant reductions in mean neointimal area for *Ccr2* ^−/−^ (**D**) and *Ndst1* ^−/−^ (**E**) transplants. Reduced intimal area is detected with donor aortic deficiency, but not with recipient deficiency (**F**, reverse transplant WT into *Ccr2* ^−/−^ or *Ndst1* ^−/−^ deficient recipient mice). Measurements reported as mean ± S.E. Arrows bracket intimal plaque, arrow heads indicates mononuclear cell infiltrates. Mag 400X.

The effects of either GAG or chemokine receptor deficiency on vascular neointimal hyperplasia (plaque) growth, was examined in mouse aortic allograft transplant models with donor aortic genetic deficiency of Ndst1 or Ccr2 (Table 1, n = 79) and wild type recipients. Excess inflammatory cell infiltration and neointimal hyperplasia (accelerated plaque growth) were detected in aortic allograft transplants at 4 weeks follow up in control, saline treated, wild type (WT) mice, using either C57Bl/6 donor to Balb/c recipient mice (Fig. 1A,E) or Balb/c donor to C57Bl/6 recipient mice (Fig. 1 C, E, n = 19). Transplantation of conditionally HS deficient *Ndst*1^−/−^ donor aorta (C57Bl/6 background) into WT recipient (Balb/c background, *Ndst1^+/+^*) mice significantly reduced plaque area (Fig. 1 B, E, n = 12), whether measured as the ratio of intimal to medial thickness (72.9% reduction, P<0.044) or by morphometric analysis of neointimal plaque area (72.4%, P<0.05), when compared to WT donor aortic transplant (Balb/c recipient) using littermate controls (Fig. 1 A, E). Similarly, transplant of *Ccr2^−/−^* donor aorta (Balb/c background) into WT (C57Bl/6 background, *Ccr2^+/+^*) recipient mice significantly reduced neointimal hyperplasia (Fig. 1 D, E, n = 23) measured as ratios of intimal to medial thickness (55.6%, P<0.040) or as total neointimal cross-section area (43.5%, P<0.021) when compared to WT (*Ccr2^+/+^*) littermate controls (C57Bl/6 recipient, Fig. 1C, E). Although Ndst1^−/−^ are on a C57Bl/6 background and Ccr2^−/−^ mice are on a Balb/c background, reductions in intimal plaque area were detected in each analysis using comparison to matched controls, e.g. WT *C57Bl/6* to Balb/c and WT Balb/c to C57Bl/6 (ANOVA p<0.027). A significant difference in total intimal plaque area was detectable for saline treated WT C57Bl/6 to Balb/c when compared to the C57Bl/6 to Balb/c controls (n = 19, mean plaque area 0.15±0.037 mm^2^ for WT Balb/c versus 0.052±0.013 mm2 for WT C57Bl/6 donor allografts, P<0.034). However, no statistically significant increase was seen on analysis of intimal to medial thickness (Fig. 1E, n = 19, p = 0.521). Measurement of the intimal to medial thickness ratios normalizes intimal plaque size to arterial medial thickness. All analyses were performed using both intimal to medial thickness ratios together with morphometric analysis of plaque area.

**Table 1:** Mouse aortic transplant model.

Donor	Recipient	Treatment Dose	Total number	Number 4 weeks
**CCR2 deficiency Studies - Allografts**			**mice**	**(Survival%)**
CCR2^+/+^ (Balb/C)	WT (C57Bl/6)	Saline	13	10
CCR2^+/+^ (Balb/C)	WT (C57Bl/6)	M-T1 0.6 ng	6	6
CCR2^+/+^ (Balb/C)	WT (C57Bl/6)	M-T1 0.6 µg	7	7
CCR2^+/+^ (Balb/C)	WT (C57Bl/6)	M-T7 0.6 ng	8	7
CCR2^+/+^ (Balb/C)	WT (C57Bl/6)	M-T7 0.6 µg	6	6
CCR2^+/+^ (Balb/C)	WT (C57Bl/6)	M-T7 6 µg	8	7
CCR2^+/+^ (Balb/C)	WT (C57Bl/6)	M3 0.6 ng	6	6
CCR2^+/+^ (Balb/C)	WT (C57Bl/6)	M3 0.6 µg	6	6
**Subtotal**			**60**	**55 (92%)**
CCR2^−/−^ (Balb/C)	WT (C57Bl/6)	Saline	10	7
CCR2^−/−^ (Balb/C)	WT (C57Bl/6)	M-T1 0.6 ng	6	6
CCR2^−/−^ (Balb/C)	WT (C57Bl/6)	M-T1 0.6 µg	6	6
CCR2^−/−^ (Balb/C)	WT (C57Bl/6)	M-T7 0.6 ng	8	7
CCR2^−/−^ (Balb/C)	WT (C57Bl/6)	M-T7 0.6 µg	11	11
CCR2^−/−^ (Balb/C)	WT (C57Bl/6)	M-T7 6 µg	12	11
CCR2^−/−^ (Balb/C)	WT (C57Bl/6)	M3 0.6 ng	6	6
CCR2^−/−^ (Balb/C)	WT (C57Bl/6)	M3 0.6 µg	6	6
**Subtotal**			**65**	**60 (92%)**
**CCR2 deficiency - Reverse Allografts**				
CCR2^+/+^ (C57Bl/6)	WT (Balb/c)	Saline	6	6
CCR2^+/+^ (C57Bl/6)	WT (Balb/c)	M-T7 6 µg	7	6
**Subtotal**			**13**	**12 (92%)**
CCR2^+/+^ (C57Bl/6)	CCR2^−/−^ (Balb/C)	Saline	7	6
CCR2^+/+^ (C57Bl/6)	CCR2^−/−^ (Balb/C)	M-T7 6 µg	6	6
**Subtotal**			**13**	**12 (92%)**
**CCR2 deficiency Studies - Isografts**				
CCR2^−/−^ (Balb/C)	CCR2^−/−^ (Balb/C)	Saline	7	6
CCR2^−/−^ (Balb/C)	CCR2^−/−^ (Balb/C)	M-T7 6 µg	7	6
**Subtotal**			**14**	**12 (86%)**
**GAG deficiency Studies - Allografts**				
NDST1^+/+^ (C57Bl/6)	WT (Balb/c)	Saline	6	5
NDST1^+/+^ (C57Bl/6)	WT (Balb/c)	M-T7 6 µg	6	6
**Subtotal**			**12**	**11 (92%)**
NDST1^−/−^ (C57Bl/6)	WT (Balb/c)	Saline	6	6
NDST1^−/−^ (C57Bl/6)	WT (Balb/c)	M-T7 6 µg	6	5
NDST1^−/−^ (C57Bl/6)	WT (Balb/c)	M-T1 15 µg	5	5
**Subtotal**			**17**	**16 (94%)**
**GAG deficiency - Reverse Allografts**				
NDST-1^+/+^ (Balb/C)	NDST-1^+/+^ (C57Bl/6)	Saline	5	4
NDST-1^+/+^ (Balb/C)	NDST-1^+/+^ (C57Bl/6)	M-T7 6 µg	4	4
**Subtotal**			**9**	**8 (89%)**
NDST1^+/+^ (Balb/C)	NDST1^−/−^ (C57Bl/6)	Saline	6	6
NDST1^+/+^ (Balb/C)	NDST1^−/−^ (C57Bl/6)	M-T7 6 µg	6	6
**Subtotal**			**12**	**12 (100%)**
**GAG deficiency - Isografts**				
NDST-1^−/−^ (C57Bl/6)	NDST-1^−/−^ (C57Bl/6)	Saline	6	6
NDST-1^−/−^ (C57Bl/6)	NDST-1^−/−^ (C57Bl/6)	M-T7 6 µg	5	5
**Subtotal**			**11**	**11 (100%)**
NDST-1^+/+^ (C57Bl/6)	WT (C57Bl/6)	Saline	7	6
NDST-1^+/+^ (C57Bl/6)	WT (C57Bl/6)	M-T7 6 µg	6	6
**Subtotal**			**13**	**12 (92%)**
**Total**			**239**	**221 (92%)**

Isograft transplants were similarly examined to assess the effects of Ndst1 or Ccr2 deficiency on isolated surgical injury. Isograft transplants using WT mouse models displayed reduced plaque areas at 4 weeks follow up, whether on a wild type C57Bl/6 or a Balb/c background. *Ndst1^−/−^* and *Ccr2^−/−^* isograft transplants demonstrated a further reduction in plaque size when compared to allografts or WT isografts. The plaque area reduction was significant when comparing *Ndst1^+/+^* allografts with isografts, but, although there is a trend toward a reduction in plaque size, there was no significant change when comparing WT isografts to genetically deficient isografts despite a trend toward a reduction in plaque size. Mean plaque area was 0.039±0.020 mm^2^ for *Ndst1^+/+^* isografts, and 0.017±0.011 mm^2^ for *Ndst1^−/−^* isografts (n = 18; p = 0.891).

These studies indicate that deficiency of either *Ndst1* or *Ccr2* in the transplanted donor aorta produced a marked reduction in intimal plaque.

### Local donor, but not systemic recipient Ndst1 or Ccr2 allograft deficiency, reduces inflammatory cell invasion and plaque growth

Local interruption of chemokine-HS GAG and chemokine-Ccr2 interactions was compared to systemic interruption, using reverse transplantations with donor WT aortic segments implanted into *Ndst1^−/−^* or into *Ccr2^−/−^* mice. Reverse transplant of Balb/c mouse aorta into *Ndst1^−/−^* (C57Bl/6 background) recipient mice did not reduce plaque size or inflammatory cell infiltrates when compared to Balb/c transplant into WT (*Ndst1^+/+^*) recipient mice (Table 1, n = 11, p = 0.468, Fig. 1F). Transplant of a WT C57Bl/6 background donor aorta into a *Ccr2^−/−^* (Balb/c background) recipient mouse also did not significantly reduce plaque size (Table 1, n = 13, p = 0.846, Fig. 1F) when compared to transplant into WT (*Ccr2^+/+^*) recipients (ANOVA p = 0.806).

Analysis of mean plaque area similarly demonstrated a trend toward a reduction in plaque area, but no significant change when comparing WT Balb/c donor aortic allograft transplant to C57Bl/6 *Ndst1^−/−^* recipient or to WT C57Bl/6 recipient transplants (28.9% reduction in intimal plaque area in the *Ndst1^−/−^* recipients, p = 0.484). No significant change was detected in WT C57Bl/6 donor to Balb/c (*Ccr2^+/+^*) or to *Ccr2^−/−^* recipient allograft transplants (Fig. 1F, 27.7% reduction in the intimal plaque area, p = 0.709).

These studies indicate that local anti-inflammatory effects of *Ccr2* and *Ndst1* deficiency inherent in the donor transplant, carried over to the recipient mouse and have predominant effects on plaque growth in the mouse aortic allograft transplant model.

### Chemokine modulating protein (CMP) treatments targeting chemokine-GAG or chemokine-CCR2 interactions reduce neointimal hyperplasia

**Figure 2:**
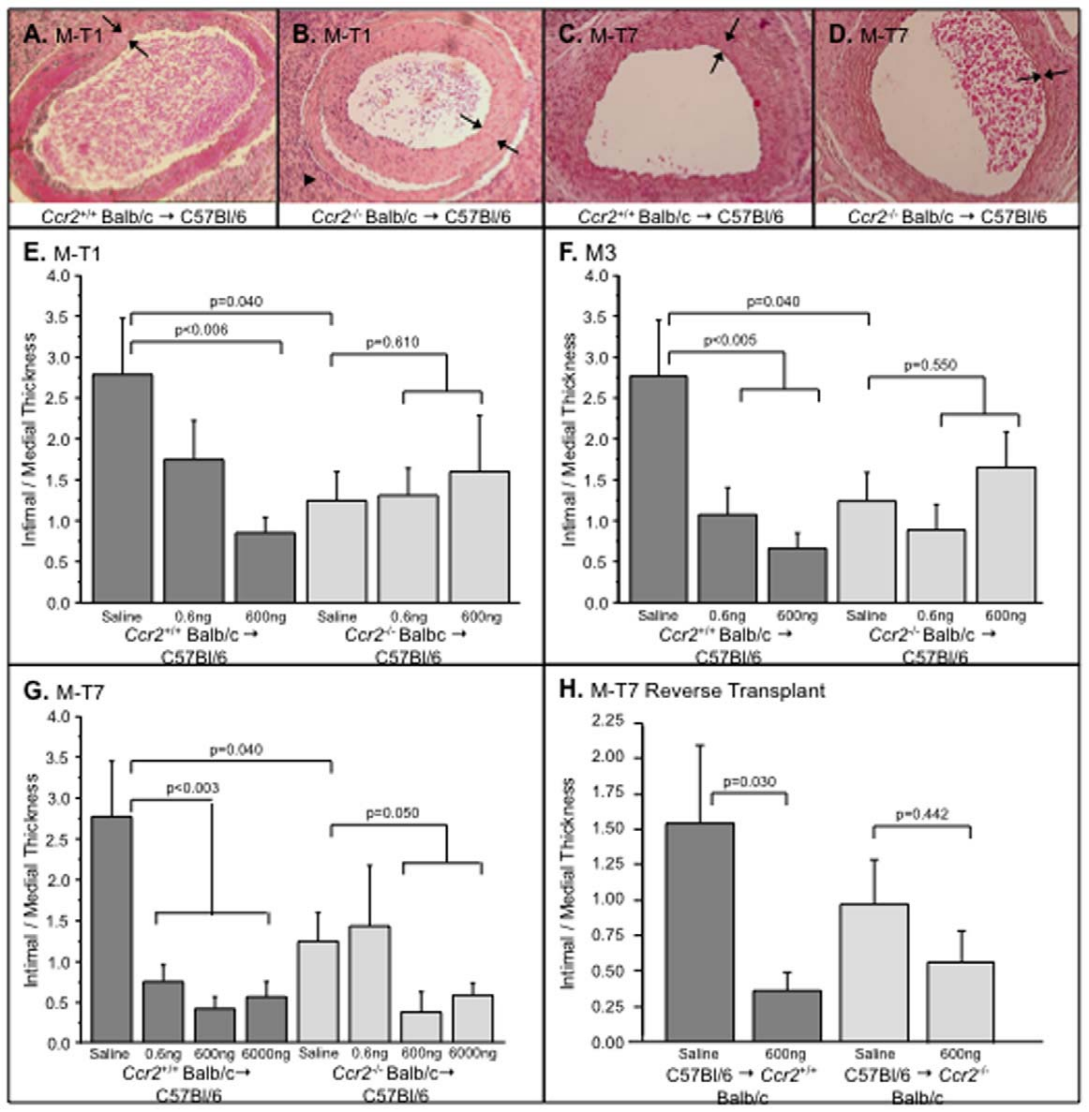
Viral chemokine modulating proteins (CMPs) significantly reduce allograft neointimal hyperplasia with donor receptor and GAG dependent specificity. H & E stained cross sections. M-T1 (A) and M-T7 (C) at 0.6 µg, or greater, doses reduced plaque in *Ccr2* ^+/+^ aortic transplants. M-T1 (B) did not inhibit intimal hyperplasia in *Ccr2* ^−/−^ donor allografts (B) whereas M-T7 did (D). Bar graphs illustrate significant reductions in intimal to medial thickness with M-T1 (E), M3 (F), and M-T7 treatment of *Ccr2*+/+ donor allografts, whereas M-T1 (E) and M3 (F) are inactive in *Ccr2* ^−/−^ donors. M-T7 did significantly reduce intimal hyperplasia in *Ccr2* ^−/−^ donor allografts (G). Reverse transplant to WT *Ccr2* ^+/+^ mice did significantly reduce intimal area, but *Ccr2* ^−/−^ recipients show only a trend toward reduction with M-T7 treatment (H). Measurements reported as mean ± S.E. Arrows bracket intimal plaque. Arrowheads point to inflammatory cell invasion. Mag. 200X.

To further examine the effects of selective blockade of chemokine-GAG interactions and chemokine-receptor interactions, we assessed the effects of three viral CMPs that target chemokine binding to either the receptor binding domain (M-T1), the GAG binding domain (M-T7), or both domains (M3). Cell invasion and intimal plaque growth were measured in the mouse aortic transplant model at 4 weeks follow up after treatment with each CMP (Fig. 2, Table 1, n = 239, including controls). To confirm the therapeutic targets for each CMP, effects were assessed after WT donor aortic transplant (*Ccr2^+/+^* and *Ndst1^+/+^*) or after transplant of *Ccr2^−/−^* (Table 1, n = 165) or *Ndst1^−/−^* (n = 74) deficient donor aorta.

Treatment with myxomaviral M-T1 infusion at a dose of 600 ng significantly reduced both intimal plaque thickness to medial thickness ratios and plaque area in WT *Ccr2^+/+^* to C57Bl/6 recipient aortic allograft transplants (Table 1, n = 26, p<0.006, Fig. 2A, E), but M-T1 did not decrease plaque growth in *Ccr2^−/−^* donor aortic allograft transplants (Table 1, n = 22, Fig. 2 B, E, p = 0.610). Infusion of M3 at similar doses reduced plaque in WT *Ccr2^+/+^* Balb/c donor to WT C57Bl/6 aortic transplants (n = 25, p<0.005, Fig. 2F), but not in *Ccr2^−/−^* donor transplants (Table 1, n = 22, p = 0.55, Fig. 2F), showing a trend toward increased plaque area. Analysis of variance (ANOVA) for M-T1 and M-T7 was significant (p<0.005 and p<0.008, respectively).

Mean plaque area paralleled the changes detected on analysis of intimal/medial thickness ratios. Specifically M-T1 and M3 reduced plaque area in *Ccr2^+/+^* donor aortic allografts (plaque area  = 0.15±.037 mm^2^ for saline, plaque area  = 0.0378±.012 mm^2^ ± for M-T1 600 ng and 0.026±.004 mm^2^ for M3 600 ng; p<0.013 for M-T1 600 ng dose and p<0.031 for M3 600 ng dose), with no significant reduction of plaque area in *Ccr2^−/−^* donor allografts (plaque area  = 0.069 mm^2^ for saline, 0.068±.03 mm^2^ for M-T1 600 ng, and 0.179±.073 mm^2^ for M3 600 ng, p = 0.36). M-T7 also reduced intimal/medial thickness ratios and plaque area after transplant of WT Balb/c donor (Ccr2^+/+^) to C57Bl/6 (Fig. 2, C; p<0.003 for 600 ng and 6 µg doses) (Table 1, n = 35, Fig. 2C, G; ANOVA, p<0.0003). Conversely, although plaque size was already reduced in the *Ccr2^−/−^* Balb/c donor allograft model, M-T7 significantly reduced intimal plaque thickness at the higher doses of 0.6 µg and 6 µg (Fisher's PLSD p<0.050, ANOVA p<0.0003) (Table 1, n = 41, Fig. 2 D, G).

Changes in mean plaque area in WT (*Ccr2^+/+^*) donor allografts again paralleled changes detected by analysis of intimal to medial thickness ratio. M-T7 reduced mean plaque area at both 600 ng and 6 µg dosing in aortic allografts (mean plaque area 0.15±.037 mm^2^ for saline, 0.045±.022 mm^2^ for M-T7 600 ng, 0.037 mm^2^±.017 for M-T7 6 µg) (ANOVA, p<0.021; p<0.027 for M-T7 600 ng dose, p<0.013 for 6 µg dose). M-T7 also significantly reduced plaque growth in *Ccr2^−/−^* aortic allografts (mean plaque area 0.069±.019 mm^2^ for saline, 0.036±.010 mm^2^ for M-T7 600 ng, 0.020±.007 mm^2^ for M-T7 6 µg, p<0.024 for 600 ng dose and p<0.003 for 6 µg dose). The maximum doses of M-T1 and M3 used for these studies at 600 ng had no demonstrated trend toward reducing plaque in the *Ccr2^−/−^* donor allograft mouse aortic transplant models (Fig. 2 E, F).

M-T7 treatment significantly reduced intimal/medial thickness ratios and plaque area in WT (*Ccr2^+/+^)* recipient reverse transplants (p<0.030), but only produced a non-significant trend toward reduced plaque in the WT C57Bl/6 to *Ccr2^−/−^* reverse transplants (Fig. 2H, p = 0.442, ANOVA p = 0.326, Table 1, n = 26). M-T7 reduced plaqu`e in the *Ccr2^−/−^* isografts, but this reduction did not reach significance, although there is a trend toward a reduction in plaque size (mean plaque area for saline treated *Ccr2^−/−^* 0.057±0.045 mm2 and for M-T7 treatment 0.013±0.006, p = 0.602).

In summary, M-T1 and M3, viral CMPs known to target chemokine-receptor interactions, inhibited plaque growth in WT aortic transplant models, but displayed reduced activity in *Ccr2^−/−^* donor to WT C57Bl/6 recipient mouse aortic transplants. M-T7, a CMP postulated to target chemokine-GAG interactions, reduced plaque growth in WT aortic transplant models, and significantly reduced plaque growth in the *Ccr2^−/−^* donor allografts, even compared to the already reduced plaque growth seen in the *Ccr2^−/−^* allograft mice.

### M-T7 inhibition of plaque growth is blocked in Ndst-1 deficient mouse aortic transplants

**Figure 3:**
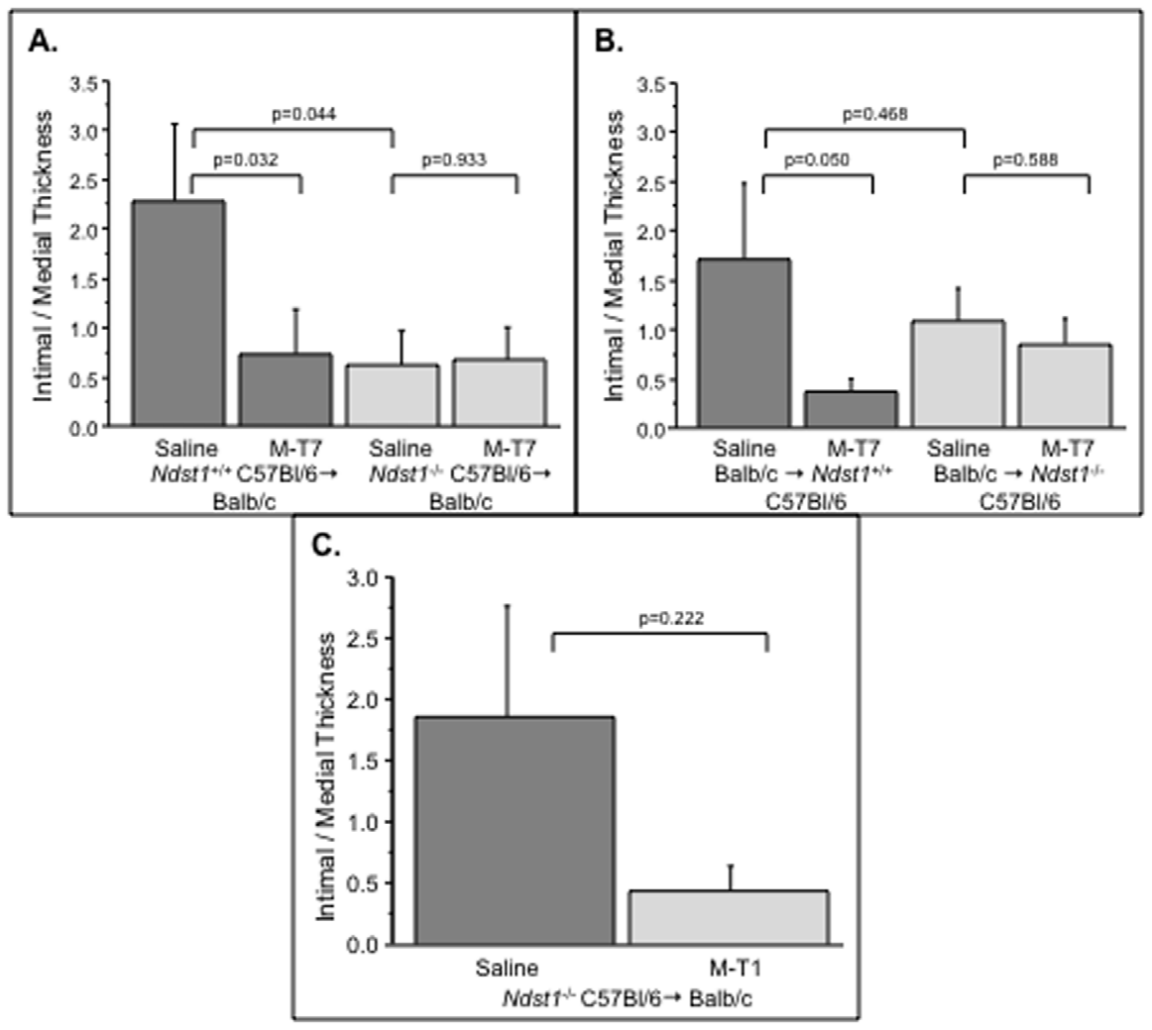
M-T7 CMP loses inhibitory activity in Ndst1^−/−^ donor allografts. Bar graphs of intimal/medial thickness ratios measured in aortic transplant cross-sections at 4 weeks follow up post transplant. M-T7 reduced plaque in WT (C57Bl/6, *Ndst1* ^+/+^) donor (P<0.032), but not in *Ndst1* ^−/−^ donor (P = 0.933) to Balb/c recipient allografts (A). In the reverse transplant of Balb/c to WT (C57Bl/6, *Ndst1* ^+/+^) M-T7 again reduced plaque (P<0.050), but not in Balb/c to *Ndst1* ^−/−^ transplants (P = 0.588). M-T1 treatment reduced plaque in *Ndst1* ^−/−^ donor aortic transplanted mice at 4 weeks follow up (C), but this trend is non significant (P = 0.222). Measurements reported as mean ± S.E.

The preceding studies indicated that M-T7 retained inhibitory, anti-inflammatory activity in *Ccr2^−/−^* aortic allograft transplants, indicating that M-T7 anti-inflammatory activity is not dependent upon Ccr2. To test whether M-T7 inhibitory action is mediated through blockade of chemokine to heparan sulfate GAG binding, we examined WT (C57Bl/6 background, *Ndst1^+/+^*) donor to WT Balb/c recipient aortic allograft transplants and *Ndst1^−/−^* donor to WT Balb/c recipient mouse aortic allograft transplants, with and without treatment with M-T7 (Table 1, n = 74). M-T7 significantly reduced intimal/medial thickness in the WT C57Bl/6 (Table 1, n = 12, p<0.032)(Fig. 3A) transplants, but did not further reduce plaque in the *Ndst1^−/−^* mouse donor allografts (Table 1, n = 12, Fig. 3A, p = 0.933, ANOVA p = 0.072).

As for the prior studies, changes in mean intimal plaque area with M-T7 treatment closely paralleled changes in intimal thickness. Mean plaque area was significantly reduced with M-T7 treatment in WT C57Bl/6 aortic donor allografts (mean plaque area  = 0.095±.032 mm^2^ for saline, 0.034±.014 mm^2^ for M-T7 600 ng, p<0.012), but not in *Ndst1^−/−^* donor allografts (mean plaque area  = 0.036±.024 mm^2^ for saline, 0.016±.009 mm^2^ for M-T7 600 ng, p = 0.306), although a trend toward a reduction is detected with M-T7 treatment. M-T7 treatment of WT C57Bl/6 donor to *Ndst1^−/−^* recipients (reverse transplants), again demonstrated a loss of M-T7 inhibitory activity (Fig. 3B, p = 0.588), whereas in the C57Bl/6 recipients M-T7 retained inhibitory activity (Fig. 3B, p<0.050).

M-T1 treatment was also tested in *Ndst1^−/−^* aortic allografts (Table 1, n = 11). While not significant M-T1 treatment did produce a trend toward reduced intimal/medial thickness (p = 0.222) and area (p = 0.245) in *Ndst1^−/−^* aortic allografts (Fig. 3C).

These studies suggest that the mode of action of M-T7 depends on fully sulfated heparan sulfate in the endothelial layer of the transplanted arteries in order to block vascular inflammation and plaque growth, consistent with M-T7 binding to the GAG binding domain C-termini of chemokines. M-T7 also did not reduce plaque significantly in the WT Balb/c to *Ndst1^−/−^* transplant model (Fig. 3B)(P = 0.548) suggesting both local and systemic effects of *Ndst1* deficiency on M-T7 activity. M-T1 also had reduced inhibitory activity in Ndst1 deficient donor allografts,

### M-T7 prolongs survival and reduces inflammatory cell invasion in renal allograft transplants

**Figure 4:**
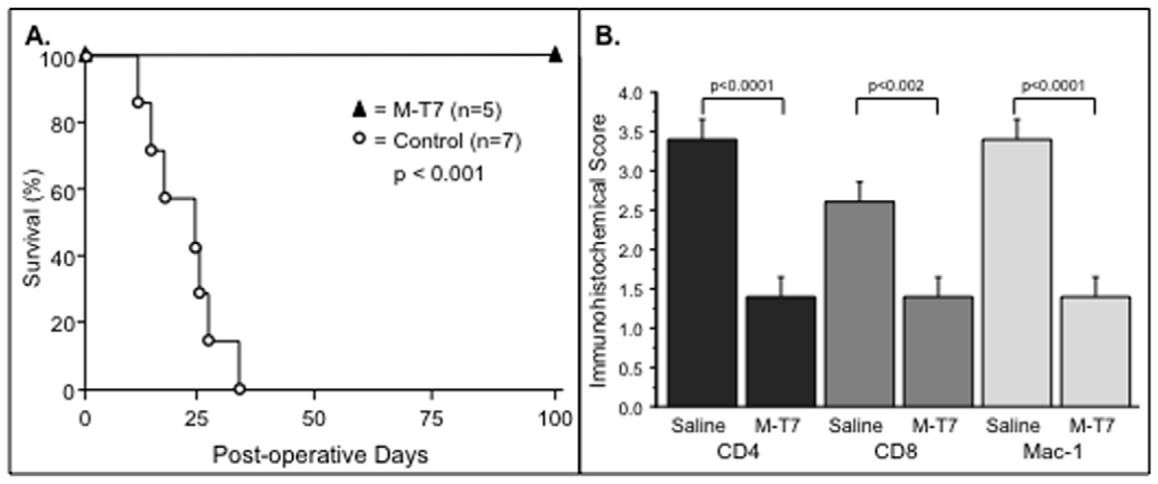
M-T7 CMP treatment of mouse renal allograft transplant recipients markedly prolongs survival. **A.** M-T7 treatment alone, with no added immunosuppressive treatment, markedly improved survival of mice to 100 days follow up after renal allograft transplant (100% survival) when compared to controls given no treatment (median survival 22.7±8.1days, 0% survival past 30 days, P<0.001). **B.** M-T7 also significantly reduced macrophage (Mac-1)(P<0.0001) and CD4 (P<0.0001), CD8 (P<0.002) positive T cell invasion as assessed by analysis histology score on immunostained cross sections taken at follow up. Measurements reported as mean ± S.E.

While the aortic allograft model provides a selective analysis of vascular changes after transplant, the vasculature in whole organ allograft transplant may or may not respond in a similar manner. Thus, renal allograft transplants were performed without immunosuppression and either saline treatment or M-T7 treatment for 10 days post transplant (n = 12). Transplant of one C57Bl/6 donor kidney into Balb/c mice with recipient mouse kidneys removed at time of transplant, causes early mortality with a median survival of 22.7±8.1 days after treatment (Fig. 4A). M-T7 treatment significantly prolonged survival to 100 days (Fig. 4A, p<0.001), the experimental endpoint for histological analysis. Immunohistochemical staining at 100 days demonstrated reduced CD4 (p<0.0001) and CD8 (p<0.002) positive T cells as well as macrophage (Mac-1, P<0.0001) invasion with M-T7 treatment (Fig. 4B, ANOVA p<0.001), despite the fact that there was no additional immunosuppressive therapy.

### Effect of deficiency or blockade of chemokine-GAG and chemokine-receptor interactions on inflammatory cell migration in aortic transplant models

**Figure 5:**
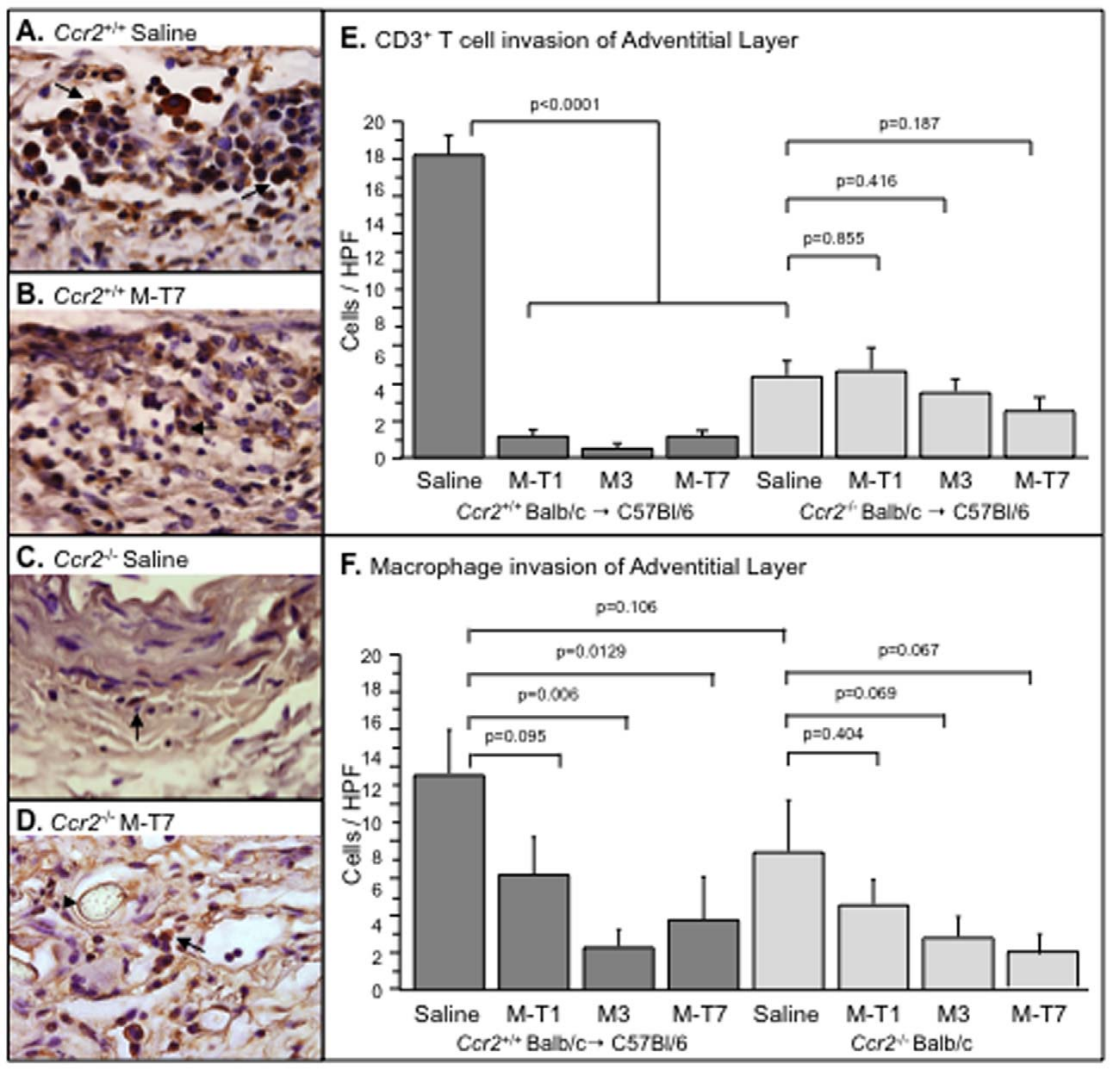
Interference with chemokine-CCR2 interactions reduces inflammatory cell invasion in aortic allografts. Immunohistochemical staining of aortic allograft transplant cross sections demonstrated increased lymphocyte (A) invasion in WT (Balb/c, *Ccr2* ^+/+^) donor aortic transplants at 4 weeks. M-T7 reduced T cell invasion in the WT Balb/c donor transplants (B). In the *Ccr2* ^−/−^ transplants T cell invasion was reduced at 4 weeks in saline treated mice (C), without further significant reduction with M-T7 treatment (D). Bar graphs demonstrate reduced T cell invasion (P<0.0001), but not macrophage (P = 0.106) in *Ccr2* ^−/−^ saline treated allografts when compared to saline treated *Ccr2* ^+/+^ allografts. M-T1, M-T7, and M3 treatment significantly reduced T cell (P<0.001)(E) and macrophage (P<0.029) (F) invasion in the WT (Balb/c, *Ccr2* ^+/+)^ donor aortic allograft transplants, but not in *Ccr2* ^−/−^ donor allografts (P = NS)(E,F). Measurements reported as mean ± S.E. Arrow heads point to brown, positively stained CD3+ T cells. Arrow points to blue stained suture seen in the top left corner of panel D. Mag 1000X.

**Figure 6:**
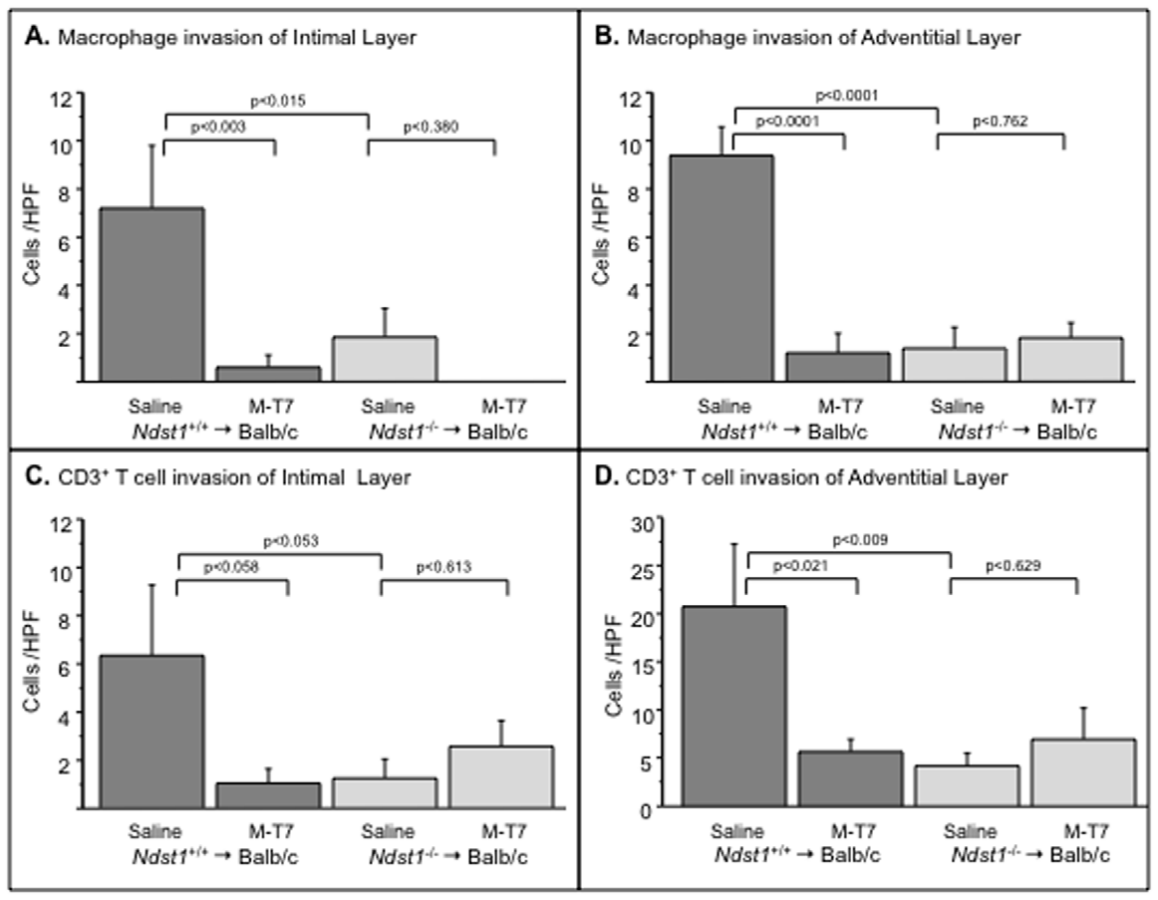
Interference with chemokine-GAG interactions blocks inflammatory cell invasion in aortic allografts. Bar graphs illustrate mean cell count per high power field (HPF) in cross sections taken from WT (C57Bl/6, *Ndst1* ^+/+^) and *Ndst1* ^−/−^ donor aortic allografts at 4 weeks follow up. Macrophage counts are reduced with M-T7 treatment in *Ndst1* ^+/+^ donor sections in the intimal (**A**, P<0.003) and adventitial layers (**B**, P<0.0001). CD3+ T cell counts are also reduced at 4 weeks follow up with M-T7 treatment in WT (C57Bl/6, *Ndst1* ^+/+^) donor allografts in both intimal (**C**) and adventitial (**D**) layers, but only adventitial counts were significantly altered (P<0021). M-T7 treatment did not reduce either macrophage or CD3+ T cell counts in *Ndst1* ^−/−^ donor aortic allografts (A,B,C,D). Measurements reported as mean ± S.E.

Comparisons of invasion of inflammatory mononuclear cells in *Ccr2^−/−^* donor allograft (to C57Bl/6 recipient) (Fig. 5) and in *Ndst1^−/−^* donor (to *Ndst1^+/+^* Balb/c recipient) (Fig. 6) with WT aortic allograft transplants was assessed by immunohistochemical cell staining of aortic cross sections. Cell invasion was reduced at 4 weeks follow up for both *Ccr2^−/−^* (Fig. 5) and *Ndst1^−/−^* (Fig. 6) aortic donor allografts with saline control treatment, when compared to WT donor controls (*Ccr2^+/+^and Ndst1^+/+^*, respectively). Selective staining for CD3-positive T cells (CD3+) demonstrated significant reductions in CD3+ T cells in saline treated *Ccr2^−/−^* donor aortic allografts (Fig. 5C, E; p<0.0001) when compared to *Ccr2^+/+^* controls (Fig. 5A, E). Compared to controls, macrophage counts were not significantly reduced in *Ccr2^−/−^* aortic allografts (Fig. 5F, p = 0.106).

Both CD3+ T cells (P<0.009) and macrophage (p<0.0001) counts in the adventitial arterial layers were reduced in *Ndst1^−/−^* donor aortic allografts (Fig. 6A–D) (ANOVA for macrophage - p<0.007 intima and p<0.0001 adventitia; ANOVA for CD3 positive T cells - p = 0.162 intima and p<0.l034 adventitia) when compared to WT Balb/c controls. Greater reductions in cell invasion in the adventitial layers (Figs. 5 and 6) were detected for both CD3+ T cells and macrophage in *Ccr2^−/−^* and *Ndst1^−/−^* donor aortic transplants than in intimal layers.

Treatment with the viral CMPs M-T1, M-T7, or M3 also significantly reduced T cell and macrophage invasion in WT C57Bl/6 donor transplants in mice (ANOVA p<0.0001 for T cell invasion and p<0.0129 for macrophage) at 4 weeks follow up (Fig. 5A,B, E, F). The inhibitory activity of all three CMPs was lost in *Ccr2^−/−^* mouse aortic donor transplant when comparing similar 600 pg and 600 ng doses for all three CMPs (Fig. 5E, F), however, M-T7 significantly reduced CD3+ T cell counts in the adventitia at the higher 6 µg dose (Fig. 5D, p<0.05; data not shown as comparable high dose M-T1 and M3 not available). A non-significant trend toward inhibition of macrophage invasion by the three CMPs was also observed in *Ccr2^−/−^* donors after treatment with the three proteins.

M-T7 was capable of inhibiting macrophage invasion into the intima (Fig. 6A, P<0.003) and adventitia (Fig. 6B, p<0.001) and CD3+ T cells (Fig. 6C, p<0.021) into the adventitia of WT C57Bl/6 mouse donor aorta transplants. In contrast, M-T7 inhibition of cell invasion was no longer evident in *Ndst1^−/−^* donor aortic allografts (Fig. 6A–D, p = NS).

These findings indicate a persistent reduction in inflammatory cell invasion at 4 weeks follow up in donor allografts with *Ccr2* or *Ndst1* genetic deficiency or after treatment of WT donor allografts with the CMPs. CMP inhibitory activity for cell invasion was reduced in *Ndst1^−/−^* and *Ccr2^−/−^* donor allografts to varying degrees, dependent upon the individual CMP tested.

### Early inflammatory cell migration in response to chemokine injection in mouse ascites is reduced with Ndst1 or Ccr2 deficiency or CMP treatment

**Figure 7:**
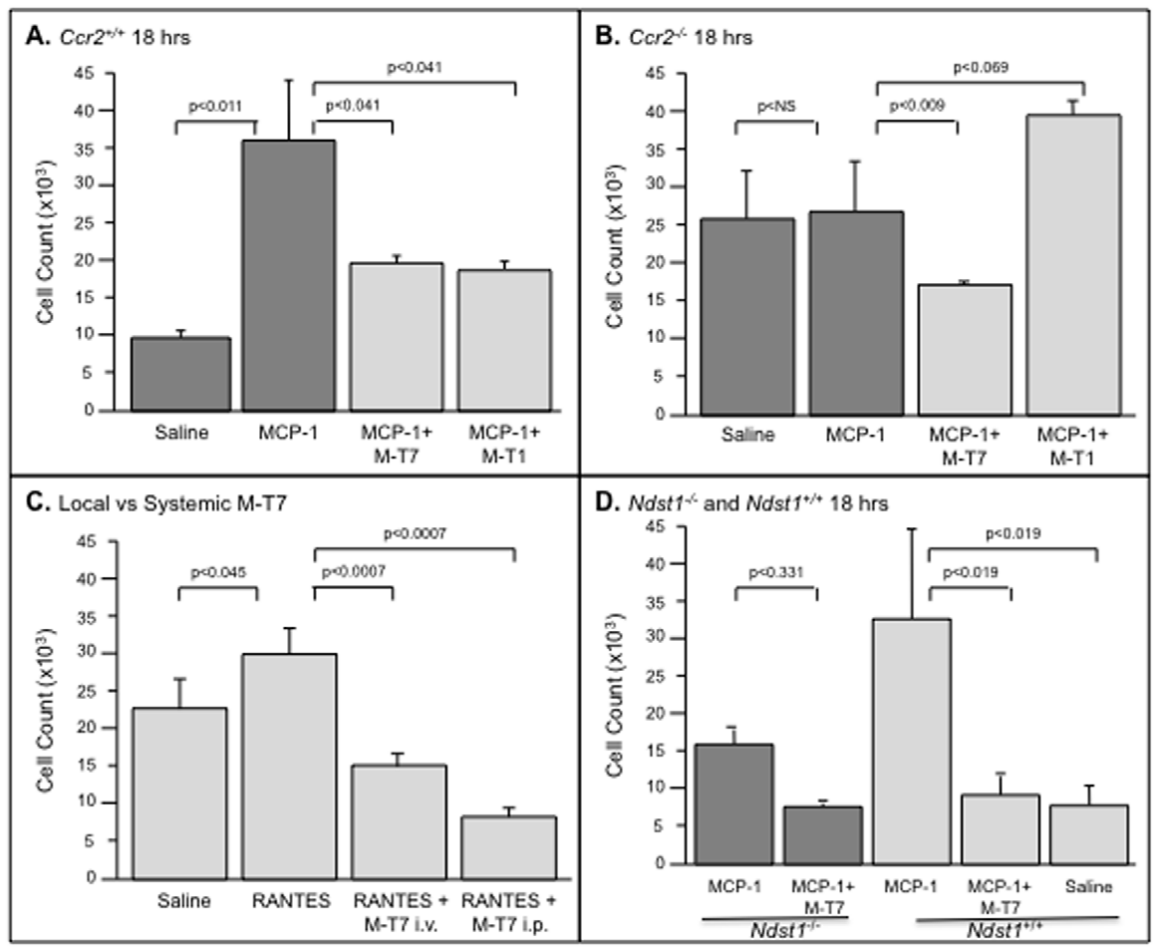
CMP treatment reduces early and local cell invasion into mouse peritoneal ascites in response to chemokine injections. FACS analysis of cell invasion counts at 18 hours into mouse peritoneal fluid in response to either MCP-1 i.p. injection (A,B,D) or RANTES i.p. injection (C). MCP-1 and RANTES both significantly increased cell migration into the peritoneal space (ascites) in WT mice (A, C, D). M-T1 and M- T7 both reduced cell invasion in WT (Balb/c, *Ccr2* ^+/+^) mice (A), but M-T7 alone was able to reduce cell invasion in *Ccr2* ^−/−^ mice (B). M-T7 also reduced cell migration into ascites in WT (C57Bl/6, *Ndst1* ^+/+^) mice (P<0.019, D) but not into *Ndst1* ^−/−^ mice (P = 0.331, D). M-T7 given either locally into the peritoneal space (I.P.) or systemically by I.V. tail vein injection was equally effective at reducing cell migration in WT mice (C). Measurements reported as mean ± S.E.

A mouse peritoneal ascites model provides a direct assessment of chemokine taxis gradient induced cell migration in the presence or absence of GAG (*Ndst1*) or chemokine receptor (*Ccr2*) expression or after CMP treatments. Observed late reductions in plaque inflammatory cell invasion and growth are predicted to be the result of early changes in inflammatory cell responses to chemokine interactions with tissue GAGs and cell receptors. Thus, early and selective chemokine-induced changes in cell migration into the peritoneum of mice were assessed after i.p. chemokine injection. Injection of each of the CC chemokines, MCP-1, RANTES, and MIP1α, increased mononuclear cell migration into the peritoneal cavity. MCP-1 and RANTES produced the most consistent changes in the mouse models and the results of injection of MCP-1 and RANTES are reported here (MCP-1 shown in Fig. 7A, B, D; RANTES shown in Fig. 7C).

Reductions in migrating cell numbers after i.p. CC chemokine injection in both *Ccr2^−/−^* (Fig. 7A, B, p = NS) and *Ndst1^−/−^* (Fig. 7D, p<0.05) mice were detected after saline control treatment in comparison to WT Balb/c or C57Bl/6 controls, respectively (*Ccr2^+/+^*, Fig. 7A, or WT *Ndst1^+/+^*, Fig. 7D). M-T1 reduced cell invasion in WT Balb/c (Fig. 7A, p<0.041), but produced a non-significant increase in cell invasion in *Ccr2^−/−^* mice (Fig. 7B, P = 0.069). M-T7 reduced cell invasion in both WT Balb/c (Fig. 7A, P<0.041) and in *Ccr2^−/−^* (Fig. 7B, P<0.009) mouse models. The capacity of M-T7 to block early chemokine mediated cell invasion was further assessed after injection via systemic (i.v.) or local (i.p.) routes (Fig. 7C). M-T7 was equally active when given by i.v. or i.p. injections indicating a local inhibitory action (Fig. 7C, p<0.0007). Conversely, M-T7 reduced cell invasion in *Ndst1^+/+^* (Fig. 7D, p<0.019), but not in *Ndst1^−/−^* mouse models (Fig. 7D, p = 0.331).

This study indicates that either *Ccr2* or *Ndst1* deficiency produced a non-significant reduction in early cell invasion in response to CC chemokine peritoneal injection. The CMPs, M-T1 and M-T7, both inhibit cell migration in response to local i.p. CC chemokine injections in WT Balb/c mice. Further, this inhibition is lost in *Ccr2^−/−^* mice for M-T1 treatment, but not for M-T7 treatment. Conversely, M-T7 inhibitory activity is lost in *Ndst1^−/−^* mice, although a trend toward a reduction is still detectable. M-T7 was equally active in WT mice whether given as a systemic (i.v.) or local (i.p.) injection.

## Discussion

With these studies we have detected equivalent blockade of allograft plaque growth after local interruption of either chemokine-GAG or chemokine-receptor interactions, whether produced by genetic deficiency or chemokine modulating protein (CMP) treatment. M-T7 treatment also markedly prolonged renal allograft survival in a mouse renal transplant model. The chemokine-GAG interaction has thus proven to be a central regulatory step in the inflammatory responses in mouse aortic and renal allograft models. We have also unexpectedly detected a predominant local effect inherent to donor aortic allografts that manifests when chemokine interactions with either GAG or Ccr2 receptor are blocked. Although chemokines are predicted to have a local effect on inflammatory responses, circulating inflammatory cells (T cells and monocytes) are believed to respond to chemokines through their receptors [5]–[9]. Chemokines and receptor interactions can also modify cellular movement into and out of the bone marrow and secondary lymphoid organs, suggesting that a systemic deficiency would also alter inflammatory cell responses post-transplant [5]–[9]. We have found a marked local effect of GAG or receptor deficiency in donor allografts, but not with deficiency in allograft recipients, suggesting a focal effect transmitted with transplant of the donor organ. These studies indicate that targeting the chemokine-GAG interaction represents a promising target for new therapeutic approaches.

Further, the CMPs targeting chemokine binding to receptor or GAG were active when given as a systemic (i.v.) dose, but these proteins were only able to block intimal hyperplasia when the donor aorta expressed the appropriate chemokine binding target. In these studies local (donor) transplant deficiency of Ccr2 and HS GAG significantly blocked inflammatory cell invasion and allograft plaque growth but systemic deficiency had no effect. Disruption of local GAG expression is expected to interrupt chemokine gradient formation and directionality of inflammatory cell migration. However, CCR2 is generally thought to be expressed on inflammatory cells and mediates responses to tissue chemokine gradients bound to GAGs. Thus, the finding of a reduction in allograft inflammation and plaque in *Ndst1* deficient mouse donors is expected, but the same reduction in *Ccr2* deficient mice was unexpected. One can postulate that either reactive ‘passenger’ inflammatory leukocytes are implanted at the time of surgery, that other cells in the donor allograft are responding, or that the activation of cells in response to CCR2 activation in the donor allograft has more extensive effects than are generally understood. Certainly others have indicated that chemokines and their receptors have much broader effects on cellular responses and that our understanding of chemokine function is as yet incomplete [9].

Prior studies in *Ccr2* deficient mice have demonstrated variable effects, with minimal effect when transplanting hearts [27] or thoracic aorta [42] into Ccr2^−/−^ mice, but a significant reduction in rejection for pancreatic islet cell transplants [27]. Although GAG deficiency on the donor endothelium is expected to alter inflammatory responses, chemokine receptor deficiency is more difficult to understand. Based upon the findings in this study, we postulate that other cells, outside of the circulating blood, and within the arterial wall such as intimal endothelium, medial smooth muscle cells, and adventitial macrophage or fibroblasts have the potential to express chemokine receptors and to respond to chemokine stimulation. Thus deficiency of CCR2 in the donor aorta can alter migration of endothelial cells, smooth muscle cells, or adventitial cells in the allograft. One prior study has detected no difference in intimal hyperplasia after either donor or recipient thoracic aortic allograft transplant into the abdominal aorta in a mouse model at 8 weeks post transplant. The differences in the results of our study for donor Ccr2^−/−^ allografts may be the result of an earlier follow up at 4 weeks, the use of a thoracic aortic segment for transplantation, or the use of Balb/c Ccr2^−/−^ strain versus a C57Bl/6 background, when compared to this prior aortic transplant study [42]. Certainly other groups have recently reported a reduction in intimal plaque after local [43] or systemic blockade of Ccr2 in vein graft transplants [44].

Conditional genetic deficiency in heparan sulfate GAG (*Ndst1^−/−^*) and genetic deficiency in the CC receptor 2 (Ccr2*^−/−^*) produced comparable reductions in vascular inflammation and plaque growth. While the studies with Ndst1 and Ccr2 deficient models demonstrated potentially equivalent roles in donor allografts, this does not constitute direct proof of selective interruption of chemokine-mediated interaction with HS GAG. Therefore we tested the effect of using CMPs derived from viruses to block either the chemokine-receptor or chemokine-GAG interactions. The targets for each CMP were further defined by comparing activity in both the WT *Ndst1^+/+^* and WT *Ccr2^+/+^* allografts and in the deficient *Ndst1^−/−^* and *Ccr2^−/−^* donor aortic allografts. With CMP treatment there was again a comparable reduction in inflammatory cell invasion and plaque growth. As predicted, M-T1 and M3 lost activity in *Ccr2^−/−^* mouse models, whereas M-T7 retained inhibitory activity. Conversely, M-T7 lost inhibitory activity in the *Ndst1^−/−^* mouse model. M-T1, however, while displaying a trend toward a reduction in both measurements of plaque area and intimal/medial thickness ratios, this reduction was not significant, suggesting that HS-GAG deficiency has wider ranging effects on chemokine interactions. Similarly, while M-T1 and M3 lost inhibitory activity for macrophage and CD3+ T cell invasion in *Ccr2^−/−^* aortic allografts, M-T7 also lost activity at comparable doses. Thus more extensive analysis of cellular responses is required to precisely determine the inflammatory cell targets for individual CMPs.

In order to define whether these cell invasion responses were the result of a directed effect against chemokine interactions, we examined chemokine mediated inflammatory cell invasion using a mouse peritoneal ascites cell migration model. In this model a chemokine gradient is induced by local injection of selected CC chemokines into the peritoneal space and early cell migration in response to chemokine injection with or without CMP treatment is then assessed. These assays allowed an analysis of early and selective CC chemokine mediated cellular responses. Inhibition of cell migration in response to the CMP treatments was again selectively lost for M-T1 in CCR2 deficient mice (Fig. 7A,B) and for M-T7 in HS-GAG deficient (Fig. 7D) mice, indicating that these inhibitory proteins do alter early cellular migration and invasion in response to chemokine activation. Further, immunostaining for CD3 positive T cells and macrophage at 4 weeks differentiated Ccr2 and *Ndst1* deficiency, with *Ccr2^−/−^* having more effect on T cell and *Ndst1^−/−^* on macrophage invasion in the adventitial layer, further suggesting the adventitia has a role in inflammatory vascular responses. Thus although a direct chemokine-receptor or chemokine-GAG interaction may not be demonstrated in the transplant models, this cell migration model does provide a direct analysis of chemokine induced cell migration. These findings therefore suggest that the loss of inflammatory responses and transplant plaque growth with M-T7 treatment is indicative of interference with chemokine-GAG interactions.

Chronic transplant rejection is generally considered to be the result of both recurrent episodes of acute rejection as well as chronic inflammation mediated damage [9]–[23], [26]–[28]. Chronic transplant scarring is often associated with vascular inflammation and occlusion at sites of scar formation and is believed due to both local and systemic or circulating cellular responses [10]–[15]. Thus circulating or systemic levels of chemokine receptors or GAGs have the potential to alter transplant rejection responses in addition to donor allograft based chemokines. Other researchers have suggested that vascular transplant alone is more representative of surgical trauma than of a true organ and/or vascular rejection response [45]. To determine whether the findings with the aortic transplant model studies are specific only to local transplanted allograft vessels or to this model alone, a mouse renal allograft model was also examined. M-T7 alone (no standard immunosuppressant treatment), reduced rejection and significantly prolonged survival. Thus, rather than simply confirming a reduction in vasculopathy and scarring, a highly significant prolongation of survival was detected. Co-treatment of M-T7 with cyclosporine has been previously reported to reduce inflammation and scarring in renal allograft transplants in a rat model [40]. However, M-T7 was not tested independent of adjuvant cyclosporine treatment in the rat renal transplant model. This current finding would suggest that M-T7, and potentially interference with chemokine-GAG interaction, provides a highly effective mechanism for blocking transplant rejection and may alter both acute and chronic rejection response. Thus one can either predict a potential separate additional activity for M-T7 or one can postulate that interference with the chemokine-GAG interaction is sufficient to block lymphocyte as well as monocyte mediated acute and chronic rejection responses. Genetic deficiency for GAG or Ccr2 has, however, not been assessed in this mouse renal transplant model and further examination of chemokine-GAG and chemokine-receptor interaction in this model is anticipated. Thus one can either predict a potential separate additional activity for M-T7 or one can postulate that interference with the chemokine-GAG interaction is sufficient to block lymphocyte as well as monocyte mediated acute and chronic rejection responses. Given the profound local effects of GAG deficiency on allograft inflammation and neointimal hyperplasia, we would postulate that M-T7 treatment and blockade of local chemokine binding to GAGs and inhibition of cell taxus is sufficient to explain the reduced inflammation and prolongation of allograft survival. Further studies will be necessary to precisely identify the mechanism of action of M-T7.

In summary, interference with chemokine-GAG interactions in donor aortic allograft transplants significantly inhibits accelerated transplant plaque growth, a reduction equipotent to blockade of chemokine-CCR2 interactions. These findings suggest that interference with inherent chemokine responses in donor allograft tissue has greater therapeutic effect. Treatment with the viral chemokine modulating protein M-T7 significantly reduced accelerated aortic allograft plaque growth. Of even greater interest M-T7 treatment given alone, with no additional immunosuppressant treatment, also markedly improved survival of mouse renal allografts. Interference with local chemokine-HS GAG interaction represents a promising new therapeutic target for vascular inflammatory disorders and transplant vascular rejection.

## Methods

### Surgical procedures

All animal surgical research protocols were performed in strict accordance with good animal practice, were approved by local laboratory animal care committees at the University of Western Ontario (London, Canada) and University of Florida (IACUC, Gainesville, USA), and conformed to the Guiding Principles for Animal Experimentation; Protocol numbers E907 and F111.Protein or control saline treatments (0.1 ml/mice) were given by intravenous (i.v.) injection (tail vein) immediately after aortic allograft transplant or immediately after renal transplant and for 9 subsequent daily tail vein i.v. injections. No increase in mortality was detected for aortic transplant models, chemokine injection for cell migration assays, mouse knock out models, or with CBP treatments. Survival rates for aortic transplant surgeries ranged from 86 to 100%. For the mouse renal transplant model, untreated animals survived 22.7±8.1 days while treated animals survived to the 100 day follow up time point.

*Ccr2* deficient (*Ccr2^−/−^*) mice (Balb/c background) were kindly provided by Dr. I. Charo [Whitehead Institute, San Francisco, CA.) [15]. The *Ndst1^−/−^* mouse strain (*Ndst1^f/f^Tek/Cre^+^*, C57Bl/6 background) and appropriate control *Ndst1^+/+^* mice (*Ndst1* ^f/f^TekCre^-^) were provided by Dr. J. Esko (Glycobiology Research and Training Center, University of California, San Diego, CA.) [4]. Mice were bred by brother sister mating in the ACVS of University of Western Ontario and at the University of Florida. All mice were genotyped prior to use. Littermate controls were used for all *Ndst1^−/−^* to *Ndst1^+/+^* comparison analyses. Wild type C57Bl/6 and Balb/c mice were obtained from JAXLabs (Bar Harbor, MN, U.S.A.).

### Aortic Allograft Transplants

In total, 239 mice had aortic allograft transplant surgery (Table 1). For analysis of effects of Ccr2 deficiency, 60 *Ccr2^+/+^* (Balb/c background) donor to WT C57Bl/6 recipient mice and 65 *Ccr2^−/−^* (Balb/c background) donor to WT C57Bl/6 recipient mice underwent aortic transplant surgery. 14 *Ccr2^−/−^* Balb/c mice also had isograft transplant and 26 mice had reverse WT C57Bl/6 donor to *Ccr2^+/+^* or *Ccr2^−/−^* recipient transplant. To assess potential effects of *Ndst1* conditional deficiency, 12 *Ndst1^+/+^* donor (C57BL/6 background) to WT Balb/c and 12 *Ndst1^−/−^* (C57Bl/6 background) to WT Balb/c aortic allograft transplants were performed. Reverse transplants, 9 *Ndst1^+/+^* (Balb/c background) to *Ndst1^+/+^* (C57Bl/6 background) and 12 *Ndst1^+/+^* Balb/c to *Ndst1^−/−^* (C57Bl/6 background) underwent aortic allograft transplant surgery to assess effects of systemic HS GAG deficiency.

All mice used for surgical studies weighed 25–30 gm and were fed normal chow for the duration of the experiments. Under general anesthetic (6.5 mg/100 g body weight Somnotrol, MTC Pharmaceuticals, Cambridge, ON, Canada) given by intramuscular (i.m.) injection, a 0.3 cm aortic segment was isolated from donor mice and transplanted into the infra-renal aorta of recipient mice for aortic allograft or isograft transplant studies, as previously described [39], [40]. The aortic transplant was inserted via end-to-end anastomosis using Sharpoint 11/0 nylon sutures (Surgical Specialties Corporation, Reiding, MA, USA) [39], [46]. Mice were followed for 4 weeks after transplant. A single infusion of each of the individual proteins M-T1, M-T7, and M3 (0, 600 pg, 600 ng, or 6 µg; 0.02, 20, 200 ng/gm body weight in 0.2 ml) was injected i.v. via tail vein immediately after transplant surgery, once aortic pulsation was again visible. Animals were sacrificed with 0.05 ml euthanyl (Bimeda- MTC Animal Health Ltd., Cambridge, ON, Canada) intramuscular (i.m.) injection. The aortic allografts develop excessive chronic arterial wall inflammation and neointimal hyperplasia with accelerated plaque growth by the end of 4 weeks.

### Orthotopic Kidney Transplantation

C57BL/6 mouse kidneys were transplanted into the abdomens of Balb/c recipients by anastomosis of the donor and recipient aortas and the donor renal vein and recipient inferior vena cava using 11–0 nylon sutures. The donor ureter was sutured to the recipient bladder using 10–0 sutures. Native kidneys were removed immediately after grafting. Graft rejection leading to death was used as the study endpoint while mice with long-term surviving grafts were euthanized at post-operative day (POD) 100 [47].

### Histological, Immunohistochemical, and Morphometric Analysis

Harvested arterial sections were processed and stained with hematoxylin and eosin (H & E) or Masson's trichrome stains as previously described [35], [37], [39], [40]. Plaque area, was measured by morphometric analysis using an Olympus CCD color video camera attached to an Olympus microscope, and the ImagePro application program calibrated to the microscope objective [39]–[41], [46]. The mean total cross-sectional intimal area or the mean intimal thickness normalized to the medial thickness were calculated for each arterial section. Allograft specimens isolated from recipient mice were cut into three sections and two histology cross sections stained per each of the three allograft section (6 sections per specimen).

For immunohistochemical staining of aortic allograft sections, formalin fixed tissues were labeled using an ABC kit (Vector Laboratories, Burlingame, USA) as per the manufacturer's protocol. Tissue sections were blocked and labeled for CD3^+^ cells using 1∶100 primary antibody (rabbit anti-mouse CD3, Abcam, Cambridge, USA), 1∶250 biotinylated secondary antibody (goat anti-rabbit IgG, Abcam) and avidin biotin complex (Vector Laboratories), as previously described [39]–[41], [46]. For renal allografts, CD4^+^ and CD8^+^ cells were detected using the biotinylated antibodies YTS 191.1.2 (Cedarlane Laboratories, Hornby, Ontario, Canada) and 53–6.7 (BD Biosciences, Franklin Lakes, NJ), respectively. Intragraft monocyte/macrophage infiltration was detected with a biotinylated anti-Mac-1 mAb (Cedarlane). Diaminobenzidine (Sigma-Aldrich, St.Louis, USA) was used for detection and sections were counterstained with hematoxylin. Positively stained cells were counted in three high power fields (HPF) areas for the intimal, medial and adventitial layers of each aortic allograft section analyzed (4–6 sections per mouse allograft specimen).

### Flow Cytometry of Inflammatory Cell Responses in Mouse Ascites

WT Balb/c and WT C57Bl/6 mice had 50 ng injections of either MCP-1, MIP-1α or RANTES (Cedar Lane, Hornby, ON, Canada) injected intra-peritoneally (i.p.) for analysis of cellular migration at 18 hours after treatment. Mice were injected with either saline, M-T1 or M-T7 at (600 ng to 6 µg) given i.v. or i.p., immediately after i.p. chemokine injection. In similar studies *Ndst1^−/−^* (C57Bl/6 background) and *Ccr2^−/−^* (Balb/c background) mice were tested after i.p. injection of chemokine with or without M-T1 or M-T7 treatment. Three mice were tested per treatment group for all mouse ascites cell migration studies. Peritoneal exudates from mice were collected with PBS peritoneal wash containing 2% FBS and treated with RBC lysis buffer (Ammonium chloride 0.15 M, potassium bicarbonate 10 mM, EDTA 0.1 mM, ph 7.4) 18 hours after i.p. chemokine injection. RBC free cells were isolated and centrifuging at 500 g for 5 min. Total cell migration count into the mouse ascites lavage was measured using FACS analysis (FACS Calibur, Becton Dickinson Canada Inc., Mississauga ON) as previously described [48].

### Source and Purification of M-T1, M-T7, and M3

Viral CBPs were expressed and purified to >90% purity on silver stained gels as previously described [39]–[41]. In brief, M-T7 was isolated from baby green monkey kidney (BGMK) cells infected with vaccinia vector expressing M-T7. M-T1 from vMyxlac-T1gpt infected BGMK cell cultures. Concentrated supernatants were fractionated by Mono QHR5/5 anion exchange chromatography followed by gel filtration chromatography using a HiLoad Superdex 200 column (AmershamPahrmacia Biotech Inc.). M3 with a six histidine tag at the carboxy terminal (M3-his) was expressed by recombinant baculovirus-infected insect cells (TN5 B1– 4, ECAAC, Xenova, Cambridge,England). Protein was purified from infected cell supernatants on Ni2-NTA resin (Qiagen). Eluted proteins were subjected to sodium dodecyl sulfate 12% polyacrylamide gel electrophoresis and visualized by silver staining and immunoblotting and judged greater than 90% pure.

### Statistical analysis

Statistical analysis was performed using Statview V5.01 (NorthCarolina, USA). The mean plaque area, ratio of intimal to medial thickness measurements, or cell counts in the intimal, medial and adventitial layers was calculated from measurements made on the three sections taken from each aortic allograft specimen isolated from each mouse at follow up (2 histology sections per section, three sections per aortic allograft; 6 sections analyzed per mouse allograft). Mean values were used for subsequent statistical analyses. Multiple group comparisons were made using analysis of variance (ANOVA) with Fishers PLSD (Protected Least Significant Difference) and additionally unpaired, two-tailed Student's T test for subgroup analysis. All bar measurements are reported as mean ± standard error (S.E.) P values less than or equal to 0.05 were considered significant.
